# Oxido-Reduction Potential as a Method to Determine Oxidative Stress in Semen Samples

**DOI:** 10.3390/ijms241511981

**Published:** 2023-07-26

**Authors:** András Balló, Péter Czétány, Kinga Székvári Busznyákné, László Márk, Nóra Mike, Attila Török, Árpád Szántó, Gábor Máté

**Affiliations:** 1 Pannon Reproduction Institute, 8300 Tapolca, Hungary; andrasballo@gmail.com (A.B.); kinga.szkvr@gmail.com (K.S.B.); drtoroka@t-online.hu (A.T.); 2 Urology Clinic, University of Pécs Clinical Centre, 7621 Pécs, Hungary; czetany.peter@pte.hu (P.C.); szanto.arpad@pte.hu (Á.S.); 3 National Laboratory on Human Reproduction, University of Pécs, 7624 Pécs, Hungary; laszlo.mark@aok.pte.hu; 4 Department of Analytical Biochemistry, Institute of Biochemistry and Medical Chemistry, University of Pécs Medical School, 7624 Pécs, Hungary; 5 MTA-PTE Human Reproduction Scientific Research Group, 7624 Pécs, Hungary; 6 Szentágothai Research Centre, Department of Physiology, Medical School, University of Pécs, 7624 Pécs, Hungary; mike.nora87@gmail.com

**Keywords:** andrology, hydrogen peroxide, infertility, lipid peroxidation, menadione, oxidative stress, oxido-reduction potential, reactive oxygen species, tert-butyl hydroperoxide

## Abstract

There are different estimates for the incidence of infertility. Its occurrence may vary from area to area, but on average, it affects 15% of couples and 10–12% of men worldwide. Many aspects of infertility can be linked to reactive oxygen species (ROS) and the process of oxidative stress (OS). The association between poor semen quality and OS is well known. Unfortunately, there is no accepted protocol for the diagnosis and treatment of OS in andrology. Oxido-reduction potential (ORP) measurement is a new method for determining the ratio between oxidant and antioxidant molecules. Currently, ORP measurement is one of the fastest and most user-friendly methods of andrological OS determination and our goals were to confirm published correlations between ORP values and sperm parameters, examine how sperm concentration influences these results, and investigate whether intracellular ROS formations are also manifested in the ORP values or not after artificial ROS induction. Intracellular ROS formations were induced by menadione (superoxide anion inducer), hydrogen peroxide, and tert-butyl hydroperoxide (lipid peroxidation inducer) treatments; sperm parameters like motility and viability were determined with an SCA Scope system, and ORP changes were recorded by the Mioxsys system. Significant correlations were noticed among the ORP, spermatozoa concentration, motility, progressive motility, and viability. Nevertheless, only the ORP value after normalization with the sperm count correlated with these parameters. Due to normalization, very low and very high sperm concentrations can give misleading results. The means of the non-normalized ORP values were almost the same. All of the applied treatments resulted in decreases in the viability, motility, and progressive motility, and interestingly, altered ORP levels were detected. In addition, it was determined that seminal plasma had a significant protective effect on spermatozoa. The elimination of seminal plasma caused higher sensitivity of spermatozoa against used OS inducers, and higher ORP levels and decreased viabilities and motilities were measured. The ORP level could be a good indicator of male OS; however, in cases of low and high sperm counts, its result can be misleading. Overall, the conclusion can be drawn that ORP determination is a suitable method for detecting intracellular ROS accumulation, but it has limitations that still need to be clarified.

## 1. Introduction

Worldwide, 15% of couples are affected by infertility, meaning that a desired pregnancy is not achieved within one year of unprotected, regular sexual intercourse [1]. The male factor is responsible for half of these cases, which means at least 30 million infertile men worldwide [2,3]. The cause of infertility cannot be identified in 25–30% of infertile men, who are subsequently classified as idiopathically infertile [4]. In contrast to idiopathic male infertility with semen parameters below the lower limit reference values according to the World Health Organization (WHO) criteria, unexplained male infertility presents with normal semen parameters [5]. One of the possible causes of idiopathic infertility is oxidative stress (OS) caused by an increase in reactive oxygen species (ROS) in the semen, seminal plasma, or spermatozoa. There may be more than 90 million infertile men worldwide, more than half of whom suffer from male oxidative stress infertility (MOSI) [6].

Increased ROS levels and reduced antioxidant protection are associated with redox imbalances, reduced sperm motility, sperm DNA damage, and with an increased risk of recurrent miscarriages and genetic diseases [7,8,9]. Spermatozoa are highly susceptible to the damaging effects of ROS due to their limited antioxidant system. These cells have very low reduced glutathione (GSH) activity and they lack many antioxidant enzymes (like catalase (CAT)), or these enzymes are present with very low activity such as superoxide dismutase (SOD) thioredoxins, peroxiredoxins, and an active mitochondrial glutathion peroxidase (GPx4) [10]. Due to the limited antioxidant response, polyunsaturated fatty acids, such as docosahexaenoic acid, which contain six double bonds per molecule, are oxidized, and the level of lipid peroxidation increases [11]. In addition, spermatozoa have a limited DNA repair system [12,13]. The ejaculate contains the spermatozoa of different maturity, leukocytes, epithelial cells, and round cells from spermatogenesis. ROS are mainly produced by leukocytes, spermatozoa with abnormal morphology, or immature spermatozoa. In addition, spermatozoa development is associated with significant ROS production, which is a major source of OS [14,15,16,17]. Physiologically, ROS are required in spermatogenesis and in the development of sperm’s fertilizing properties: chromatid compaction during maturation, motility, chemotaxis, capacitation, hyperactivation, acrosome reaction, and binding to the zona pellucida, inducing sperm–egg fusion [18,19,20,21].

Several direct and indirect tests exist to detect OS, focusing on ROS formation, lipid peroxidation production, and total antioxidant capacity (TAC). In andrology, a novel test is the oxido-reduction potential (ORP) measurement, where the ORP value indicates the ratio between oxidant and antioxidant molecules. The ORP is assessed by the Mioxsys test, which measures electron transfer from antioxidants to oxidants using a low-voltage reducing current. The data obtained indicate the oxidant and antioxidant activity in a sample: high ORP levels indicate increased oxidant activity, and thus, a state of OS [22].

For the time being, there are no infertility guidelines that recommend routine ROS measurement, and there is debate about who should be tested for OS. Asthenozoospermia in semen samples is likely a marker of ROS [23]. Hyperviscosity is also indicative of increased OS, which may be attributed to increased malondialdehyde (MDA) levels. The increased presence of leukocytes or round cells may raise the need for OS testing; leukocytes can be signs of inflammation in the reproductive tract. Abnormal sperm morphology due to cytoplasmic debris also correlates with high ROS levels. The identification and treatment of OS is also warranted when using assisted reproductive techniques, as many of the sperm preparation and treatment methods used in ART can induce OS [24].

In clinical practice, the ORP determination can currently be the fastest, most user-friendly method that does not require special expertise to determine the OS status of semen samples. However, the results obtained with the method are quite controversial. There is agreement in the literature that in the case of samples with high ORP results, poorer sperm functional parameters are experienced in motility, progressive motility, and viability [25]. In a recent study by Castleton et al. [26], increased ORP levels and altered sperm parameters (decreased motility, decreased morphology, and increased DNA fragmentation) showed significant correlations, but these correlations were mainly influenced by the sperm concentrations (the ORP result has to be normalized with the cell concentration: the higher the concentration, the lower the normalized ORP). The results may be distorted in the case of the lower and upper extremities. Their conclusion was that this method is not enough to determine sperm OS due to the great impact of the sperm concentration on final results. The aims of our study were to (i) confirm the published correlations between the ORP and sperm parameters, (ii) examine how the sperm concentration influences these results, and (iii) investigate whether intracellular ROS formations are also manifested in the ORP level or not. For the last point, three different types of OS were applied, and all of the used chemicals have different modes of action to determine the sensitivity of the Mioxsys system against intracellular ROS formation. Namely, menadione exposures were used to trigger superoxide anion (O_2_^●−^) formation in sperm cells, hydrogen peroxide (H_2_O_2_) exposures were applied as a direct OS source, and tert-butyl hydroperoxide (t-BuOOH) treatments were performed to cause the peroxidation of lipids.

## 2. Results

The semen analyses of the investigated period were divided into two groups based on the counted cell number: oligozoospermic (lower than 16 M/mL sperm) and non-oligozoospermic (higher than 16 M/mL sperm). In comparison to the non-oligozoospermic group, oligozoospermic samples had lower motility (25.8 ± 14.1% vs. 37.4 ± 17.8%, *p* < 0.1%), lower progressive motility (19.7 ± 13.1% vs. 28.8 ± 15.8%), and lower viability (60.5 ± 8.7% vs. 67.4 ± 11.6%, *p* < 0.1%), but a higher normalized ORP value (8.5 ± 13.9 mV/10^6^/mL vs. 1.0 ± 0.9 mV/10^6^/mL, *p* < 1%) and DNA fragmentation (20.5 ± 10.6% vs. 16.8 ± 10.0%, non-significant). Interestingly, before normalization with the spermatozoa concentration, the difference in the ORP value between the two groups was not considerable (38.5 ± 16.6 mV vs. 40.6 ± 25.5 mV, non-significant). As a function of concentration, a difference can be seen in the ORP value only with the normalized values (Table 1).

**Figure 1:**
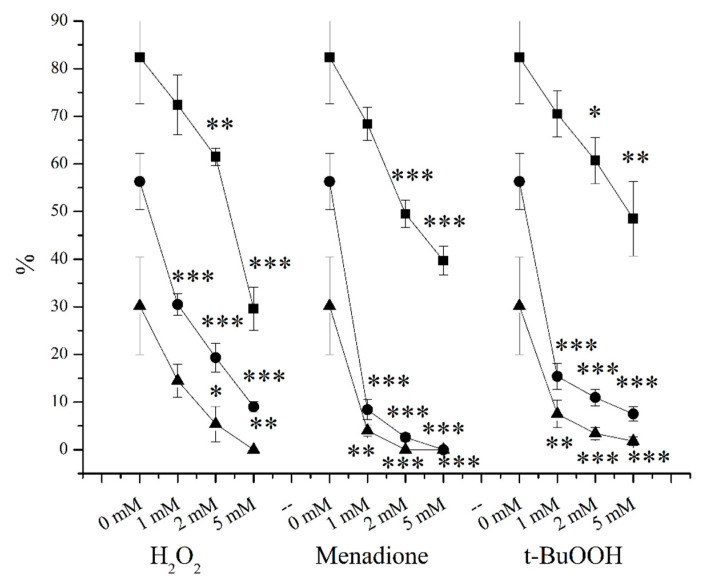
Exposure of raw semen samples for 2 h at 37 °C with H_2_O_2_, menadione, and t-BuOOH. Viability (▪), motility (●), and progressive motility of sperm cells (▲). * *p* < 5%; ** *p* < 1%; *** *p* < 0.1%. *p* values were calculated via the Mann–Whitney test.

All of the applied treatments induced concentration-dependent decrement in the measured parameters (Figure 1). Raw semen samples with 1 mM, 2 mM, and 5 mM H_2_O_2_ exposure for 2 h resulted in 12.1%, 25.3%, and 64.1% decrements in the viability of sperm cells, 45.9%, 65.7%, and 84.1% decrements in their motility, and 52%, 82.2%, and 100% decrements in their progressive motility, respectively (the alterations are described as % of decrements). Then, 1 mM, 2 mM, and 5 mM menadione induced 17%, 40%, and 51.9% decrements in the viability of sperm cells, 85.1%, 95.4%, and 100% decrements in their motility, and 16.8%, 100%, and 100% decrements in their progressive motility, respectively. Finally, 1 mM, 2 mM, and 5 mM treatments with t-BuOOH caused 14.5%, 26.4%, and 41.2% decrements in the viability of sperm cells, 72.7%, 81.6%, and 86.7% decrements in their motility, and 75.2%, 88.8%, and 94.1% decrements in their progressive motility, respectively (Figure 1). DMSO was applied in quite a high concentration, it seems to not have had any detectable negative effects but additive effects cannot be excluded.

**Figure 2:**
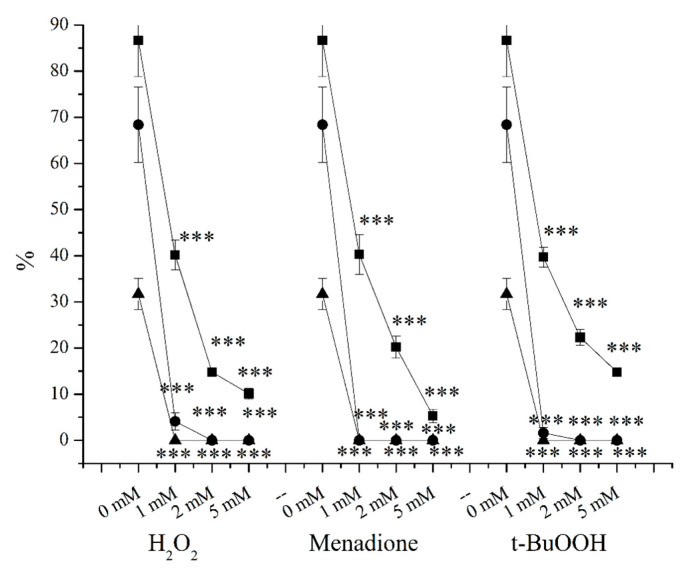
Exposure of plasma-free sperm cells for 2 h at 37 °C with H_2_O_2_, menadione, and t-BuOOH. Viability (▪), motility (●), and progressive motility (▲). *** *p* < 0.1%. *p* values were calculated via the Mann–Whitney test.

In the case of centrifugated and washed sperm cells, the same phenomenon was observed (Figure 2). Samples with 1 mM, 2 mM, and 5 mM H_2_O_2_ exposure for 2 h resulted in 53.7%, 83%, and 88.4% decrements in the viability of sperm cells, 94.1%, 100%, and 100% decrements in their motility, and 100%, 100%, and 100% decrements in their progressive motility, respectively. Then, 1 mM, 2 mM, and 5 mM menadione induced 53.6%, 76.8%, and 93.9% decrements in the viability of sperm cells, 100%, 100%, and 100% decrements in their motility, and 100%, 100%, and 100% decrements in their progressive motility, respectively. Finally, 1 mM, 2 mM, and 5 mM treatments with t-BuOOH caused 54.3%, 74.3%, and 83% decrements in the viability of sperm cells, 100%, 100%, and 100% decrements in their motility, and 100%, 100%, and 100% decrements in their progressive motility, respectively (Figure 2).

**Figure 3:**
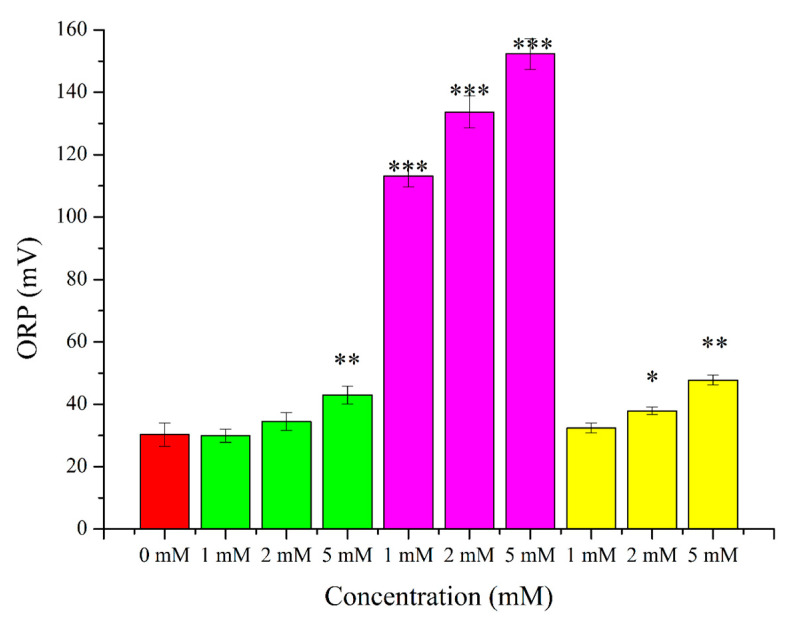
Exposure of raw semen samples for 2 h at 37 °C with H_2_O_2_ (green columns), menadione (purple columns), and t-BuOOH (yellow columns). The oxido-reduction potentials were determined by a Mioxsys Analyzer, and results are given as mV. * *p* < 5%; ** *p* < 1%; *** *p* < 0.1%. *p* values were calculated via the Mann–Whitney test.

After the treatment of semen samples for two hours with 1 mM, 2 mM, and 5 mM H_2_O_2_, 1 mM, 2 mM, and 5 mM menadione, and 1 mM, 2 mM, and 5 mM t-BuOOH, 0.98-fold, 1.13-fold, 1.42-fold, 3.73-fold, 4.41-fold, 5.02-fold, 1.06-fold, 1.25-fold, and 1.57-fold increments could be observed, respectively (Figure 3). The results are given in mV and were normalized with the sperm concentration (10^6^).

**Figure 4:**
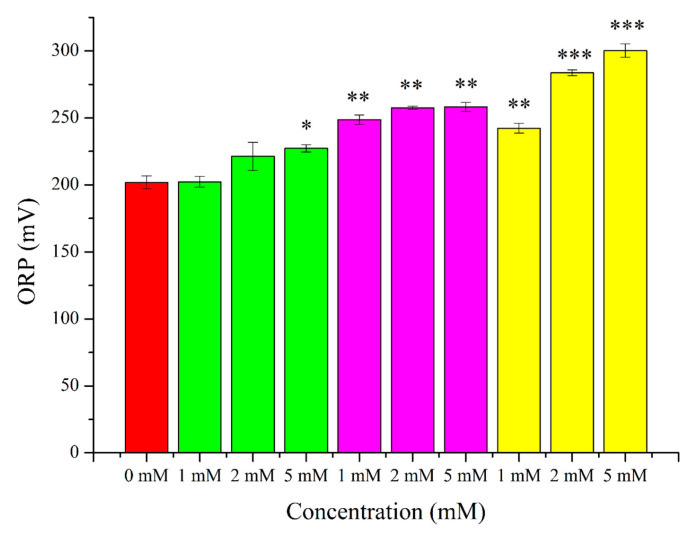
Exposure of plasma-free sperm cells for 2 h at 37 °C with H_2_O_2_ (green columns), menadione (purple columns), and t-BuOOH (yellow columns). The oxido-reduction potentials were determined by a Mioxsys Analyzer and results are given as mV. * *p* < 5%; ** *p* < 1%; *** *p* < 0.1%. *p* values were calculated via the Mann–Whitney test.

After the treatment of plasma-free sperm samples for two hours with 1 mM, 2 mM, and 5 mM H_2_O_2_, 1 mM, 2 mM, and 5 mM menadione, and 1 mM, 2 mM, and 5 mM t-BuOOH, 1.00-fold, 1.09-fold, 1.12-fold, 1.23-fold, 1.27-fold, 1.27-fold, 1.20-fold, 1.40-fold, and 1.48-fold increments could be observed, respectively (Figure 4). The results are given in mV and were normalized with the sperm concentration (10^6^). The y-axis of Figure 3 differs from that of Figure 4 because the elimination of seminal plasma and its replacement with PBS resulted in a huge increase in the initial ORP value.

## 3. Discussion

### 3.1. OS in Andrology

The redox equilibrium between the oxidative factors and the antioxidant components of a biological system is a crucial coefficient of its homeostasis. The state when the effect of the oxidative elements is overwhelming the protective antioxidant mechanisms is known as OS. Conversely, we can also talk about reductive stress. The main contributing elements of OS are ROS, which consists of free radicals (e.g., O_2_^●−^ and hydroxyl radical) and nonradical reactive species (e.g., H_2_O_2_ and singlet oxygen). Nonradical reactive species show weaker reactivity compared to free radicals, which have unpaired electrons on their outer electron shell. Consequently, they show high reactivity with biomolecules (lipids, proteins, nucleic acids), and thus, the alteration of these molecules can lead to cellular dysfunction, and even apoptosis or necrosis of the cell [19,27]. OS have been identified as a cornerstone of the pathomechanism of several human diseases, such as neurodegenerative diseases (Alzheimer’s and Parkinson’s disease, amyotrophic lateral sclerosis, etc.) [28,29,30], cardiovascular diseases (hypertension, heart failure, atrial fibrillation, etc.) [31,32,33], diabetes mellitus [34], obesity [35], chronic kidney disease [36], malignant tumors (gastrointestinal, lung, prostate, breast, etc.) [37,38,39,40], and cataracts [41]. The probable role of OS in the development of male infertility and the protective properties of the seminal plasma were presumed in 1994 by Aitken [42,43]. OS can trigger the impairment of sperm function (e.g., energy production, motility), and DNA fragmentation can occur and can even lead to apoptosis [44]. Recently, OS-induced male infertility has widely been researched and discussed by most clinical guidelines (European Association of Urology (EAU), European Academy of Andrology (EAA), European Society for Human Reproduction and Embryology (ESHRE)), but antioxidant treatment is still controversial, since there are no randomized controlled trials supporting the use of them [45,46,47].

The effects of various environmental factors on ROS burden of semen are well-established: smoking, alcohol, electromagnetic radiation, air pollution, obesity–metabolic syndrome, varicocele, etc. Various mechanisms could be revealed in the background. Tobacco smoke contains high levels of ROS, and a higher ratio of leukocytospermia was observed among smoking men [48]. Chronic use of alcohol can result in increased seminal OS caused particularly by enhanced lipid peroxidation, while the intratesticular androgen synthesis decreases [49,50]. Electromagnetic radiation (e.g., cell phones, laptops) not only alters the microarchitecture of testicular tissue and deteriorates the sperm count, morphology, motility, and viability, but induces ROS formation (as a result of a non-thermal effect) and DNA fragmentation as well [51,52,53,54]. In a study by Zhang et al., bad air quality, particularly SO_2_ exposure, correlated with a higher MDA level as a marker of lipid peroxidation level, indicating OS [55]. Li et al. showed that methyl tert-butyl ether (MTBE), an agent emitted by vehicles, has a direct cytotoxic effect on rat spermatogenic cells via ROS production [56]. The increased fatty acid oxidation in mitochondria and peroxisomes in obesity can lead to increased ROS production [57]. The role of OS in the contribution of varicocele to male infertility has been known for a long time. The primary mechanism is the following: the venous stasis in the pampiniform plexus causes an increased temperature, and the consecutive heat stress, hypoxia, and chronic inflammatory response lead to increased ROS production, deteriorating sperm quality [58,59,60]. Increasing seminal OS is connected to the aging process [61].

Above all, ROS plays a key role in the regulation of several physiological processes of spermatozoa maturation and fertilization. Capacitation is a complex action making the spermatozoa capable of hyperactivation (high-amplitude, non-linear movement helping the penetration of the cumulus oophorus), zona pellucida recognition, and acrosome reaction (release of digestive enzymes helping the penetration of cumulus cells and zona pellucida) [19]. All of these are essential for successful fertilization. The complete mechanism of capacitation is not clearly known, but it involves the activation of c-AMP-dependent molecular pathways [62] and a decrease in the cholesterol/phospholipid ratio in the cell membrane [63], influencing membrane fluidity and extensive tyrosine phosphorylation [16,64].

In andrology, there are several methods for measuring ROS and their oxidized products. These methods can be direct or indirect assays. Direct assays include chemiluminescence, the nitroblue tetrazolium test, cytochrome c reduction, flow cytometry, electron spin resonance, and xylenol orange-based assay. Indirect methods are the myeloperoxidase test, redox potential measuring, lipid peroxidation levels, chemokines, antioxidants, and antioxidant enzymes, and DNA damage level measurement [65].

Chemiluminescence assay detects oxidized end products. Luminol and lucigenin probes can be used with the assay. Luminol oxidizes at the acrosomal level. Lucigenin is oxidized at the extracellular level. In the presence of H_2_O_2_, a heterogeneous group of sperm peroxidases, which causes the intracellular deoxygenation of luminol. This deoxygenation is the reason for the luminol-mediated chemiluminescence signal in spermatozoa. The free radical combines with luminol to produce a light signal that is converted to an electrical signal (photon) by a luminometer. Lucigenin is oxidized by O_2_^●−^ at the extracellular level [66,67,68]. Luminol is very sensitive and reacts with different types of ROS at neutral pH, can also measure the global level of ROS since it can measure both extracellular and intracellular ROS under physiological conditions, and it is easy to use. The last chemiluminescent signal is produced by every spermatozoon because it is the combined sum of the partial signals [69,70].

Another accepted method for seminal OS analysis is the ORP determination, and this method is basically known as the Mioxsys system. It reads the electron flux voltage from seminal redox reactions with a galvanostatic-based sensor, and the displayed result (mV) is a static ORP (sORP), which is a measure of the overall balance between oxidants and antioxidants, providing a comprehensive measure of OS. After the measurement, the sORP value has to be normalized with the cell concentration (10^6^) [25,65,71]. Little published information is available on the exact operating principle of the measurement, and the published results are controversial. In some studies, there is a significant correlation between the ORP values and the parameters of spermatozoa or IVF outcomes. In a recent paper by Henkel et al. [72], increased ORP levels were manifested in a reduced blastocyst formation rate, reduced implantation rate, and reduced live birth rate. In addition, the ORP was correlated with the male age, sperm DNA fragmentation, sperm concentration, motility, and morphology. Within the framework of a retrospective analysis of data from our institute, the same phenomenon has been experienced regarding correlation between the ORP and the properties of spermatozoa. During this analysis, the influence of the sperm concentration on other parameters was investigated. In the oligozoospermic group, reduced motility, progressive motility, and viability, and higher DNA fragmentation and normalized ORP values were recorded. Interestingly, without normalization, the ORP value did not differ significantly between the two analyzed groups (Table 1). This raises the question of how much the correction with the cell count distorted the ORP result. For instance, during our measurements, the samples with more than a 50 × 10^6^ sperm/mL sperm count always had a low ORP level (<1.36), while the samples with less than a 10 × 10^6^ sperm/mL sperm count always had a high ORP level (data not presented). Is it necessary for a severe oligozoospermic sample to always have a high ORP value? Or does a good normozoospermic sample always have a low ORP level and the presence of OS is excluded? In the paper by Agarwal et al. [65], in addition to viscous samples, severe oligozoospermia (<2 × 10^6^ sperm/mL)) is mentioned as one of the limiting factors for the reliability of the ORP value. Based on our data, the problematic limit of the sperm count—where the ORP value is distorted—is <10 × 10^6^ sperm/mL. In the following phase, we wanted to investigate whether the sperm count affected the results so much because the measurement is suitable for determining the full range of intracellular ROS and the more cells there are, the more intracellular ROS there are. For this purpose, treatments with external OS inducers were used.

In our study, three different types of OS were applied, and all of the used chemicals have different modes of action to determine the sensitivity of the Mioxsys system against intracellular ROS formation. Namely, (i) menadione exposures were used to trigger O_2_^●−^ formation in sperm cells, (ii) H_2_O_2_ exposures were applied as a direct OS source, and (iii) t-BuOOH treatments were performed to cause the peroxidation of lipids.

### 3.2. O_2_^●−^ Induction by Menadione and Its Significance

The main endogenous sources of ROS and O_2_^●−^ are the electron transport chain (ETC) in the mitochondria and the membrane-associated nicotinamide adenine dinucleotide phosphate (NADPH) oxidase. In addition, ROS are produced by a cytochrome P450 and its NADPH-dependent reductase in the endoplasmic reticulum, the xanthine oxidase, the NADH- and NADPH-consuming electron transport system in the nuclear membrane, and the peroxisomes. In ETC, electron slippage to molecular oxygen can occur from the NADH-ubiquinone-reductase complex and from reduced ubiquinone itself. NADPH oxidase is present in the cell membrane of the neutrophil granulocytes and is responsible for the release of ROS during “respiratory burst”, aiming to eliminate pathogens. The cytochrome P450 has a role in the hydroxylation of xenobiotics and the endogenous substrates of the cell (fatty acids, steroids), and therefore, it is oxidized. The oxidized enzyme itself and the reduced form of its reductase could be a source of slipping electrons. The xanthine oxidase catalyzes the oxidation of hypoxanthine to xanthine and the further oxidation of xanthine to uric acid, while molecular oxygen is reduced to O_2_^●−^ and H_2_O_2_. Peroxisomes contain a wide range of oxidative enzymes and are responsible for a key role in lipid metabolism [73,74,75]. In the semen, immature germ cells and leukocytes are the most significant contributors to OS [23,44]. In spermatic cells, the mitochondrial ETC and the membrane-associated NADPH oxidase are the main intrinsic sources of ROS [19,76]. Menadione is a O_2_^●−^ generator that induces the formation of O_2_^●−^ within the cell through single-electron transfer reactions. In addition, the metabolism of menadione by single-electron reducing enzymes, such as mitochondrial NADH-dependent ubiquinone oxidoreductase and microsomal NADPH-dependent cytochrome P450 reductase, creating an unstable semiquinone radical, and its reverse oxidation also produces O_2_^●−^ if molecular oxygen is present [77]. O_2_^●−^ is a highly reactive radical; however, its reactivity strongly depends on its medium. It has a relatively high reactivity in hydrophobic environments, while its reactivity is low in hydrophilic conditions. In an organic solution, O_2_^●−^ is a strong base, while in an aqueous solution, it has both reducing and weak oxidizing properties. In the latter case, it can form conjugates with weak acids, e.g., the hydroperoxyl radical (protonated O_2_^●−^, HO_2_^●^), which turns into H_2_O_2_ in three steps during disproportionation. O_2_^●−^ is able to reduce or oxidize transition metals, such as iron. Due to its reducing property, it reduces cytochrome c in the respiratory chain, and due to its weak oxidizing property, it oxidizes polyphenols, tocopherol, ascorbate, and thiols (e.g., cysteine); it can also inactivate the antioxidant enzyme CAT. In addition, due to its reducing properties, it is able to destroy the Fe-S clusters of proteins through the reduction of Fe(III). The released iron can cause further damage (e.g., through the Fenton reaction). Furthermore, the cells are not able to recycle the reduced iron thus formed, an iron deficiency occurs, and the resynthesis of the Fe-S cluster is also disturbed [78]. In the literature, the effects of menadione-triggered ROS formation on sperm cells are well discussed. In the study by Aitken et al. [79], 0–50 µM menadione treatments for 15 min induced concentration-dependent decreases in the motility and progressive motility and significant increments in the intensity of applied ROS probes (dihydroethidium, dichlorodihydrofluorescein, nitroblue tetrazolium, MitoSox Red, lucigenin, and luminol). In another study, Zhu et al. [80] investigated the OS-related properties of menadione-exposed boar sperm cells and a significant accumulation of ROS, depletion of the GSH levels, and decreases in the mitochondrial membrane potential, membrane integrity, and ATP level were observed. Similar results have been published several times [81,82].

### 3.3. Hydrogen Peroxide

All systems that produce O_2_^●−^ also produce H_2_O_2_ as a result of the disproportionation reaction. This transformation can occur non-enzymatically through disproportionation, or enzymatically by SODs. Another important source of H_2_O_2_ is the peroxisome of eukaryotic cells. Due to the deamination of amino acids by amino acid oxidases and the oxidation of glucose to δ-gluconolactone by glucose oxidase, peroxisomes are considered a significant source of H_2_O_2_ [73]. Only O_2_^●−^ and H_2_O_2_ can serve as both oxidizing and reducing agents, and their reactivity is highly limited in aqueous media. However, unlike O_2_^●−^, it can penetrate almost all biological membranes (since it has no charge), and thus, it can induce oxidative damage far from the place of origin [83]. It can create adducts with many components of biological systems, and the resulting hydrogen-bonded chelates and H_2_O_2_ carriers increase the diffusion distance even more. One of the most effective adduct formers is histidine. L-histidine has been shown to increase H_2_O_2_-induced chromosomal aberrations eightfold [84]. H_2_O_2_ can damage the cysteine and methionine side chains of proteins. As a result, e.g., the sulfenyl group (SO^−^), sulfonyl group (SO_2_^−^), and sulfinyl group (SO_3_^−^) can appear on cysteine side chains, and methionine sulfoxide can be formed from methionine [78]. In addition, the importance of H_2_O_2_ in terms of OS lies in the formation of the extremely reactive ^●^OH. Numerous studies can also be found in the case of H_2_O_2_ treatment. In each of these, a decrease in the viability of sperm cells, deterioration of the kinetic parameters, reduced capacitation and penetration ability, and increased lipid peroxidation can be observed [85,86].

### 3.4. Lipid Peroxidation Induction by t-BuOOH

Lipid peroxidation is the oxidative damage of unsaturated lipids. Polyunsaturated fatty acids are primarily affected, but monounsaturated or even saturated fatty acids and membrane cholesterol can also be oxidized. Free radicals are primarily responsible for starting the process (primarily ^●^OH, since O_2_^●−^ can only pass through biological membranes through channels); however, lipid peroxidation is also enzymatic via cyclooxygenases, lipoxygenases, or cytochrome P450 monooxygenases as a natural process of the phospholipid cycle can take place [87]. On the one hand, free radicals are able to connect to fatty acids by breaking the unsaturated bond, and on the other hand, they are able to remove a hydrogen atom from the methylene group, creating a lipid radical. The resulting lipid radical reacts with an oxygen molecule, and a peroxyl radical is formed. Then, a chain reaction-like process starts (Figure 2): the peroxyl radical abstracts hydrogen from another fatty acid, creating a new lipid radical, which itself turns into a lipid peroxide (lipid hydroperoxide) [78,88,89]. Although lipid hydroperoxide is not a radical molecule, it can serve as a starting point for the formation of other reactive radicals (e.g., peroxyl radical or epoxy-allyl-peroxyl radical) [90]. The lipids that make up the membranes are amphipathic, containing a polar head part and a hydrophobic tail part. As a result of lipid peroxidation, the hydrophobicity of the tail part increases, as a result of which the biological functions of the membranes can be damaged (e.g., transport processes, respiration, cell wall synthesis) and their permeability increases [91]. t-BuOOH is an organic lipid hydroperoxide analog that induces lipid peroxidation as an externally applied prooxidant, thus making it suitable for studying the process. The toxicity of t-BuOOH is based on the formation of the butoxyl radical through a Fenton-type reaction [78,88]. Spermatozoa are extremely susceptible against ROS because they are poor in antioxidant enzymes such as SOD, GPx, thioredoxins, and peroxiredoxins, and the CAT enzyme is completely absent [92]. As a consequence of poor antioxidant activity and the high ratio of polyunsaturated fatty acids of the plasma membrane of sperm cells, (these polyunsaturated fatty acids are one of the main targets of ROS) lipid peroxidation can occur [93]. In an in vivo study, male Wistar rats were IP injected with 0–40 μM t-BuOOH. The injections were administered for 5 consecutive days per a week for 60 days. After euthanization, tissue of the testes and epididymis was analyzed [94]. Reduced sperm concentration and motility, increased MDA levels of both the spermatozoa and testes tissue, and decreased activities of SOD and GSH were noticed. Kumar and Muralidhara [95] published similar results, with the difference that more antioxidant enzymes were tested and differences were found. Beyond these, significant DNA oxidation was also found by Wu et al. [96].

### 3.5. Effects of OS-Inducers on Parameters of Spermatozoa and ORP Levels

In agreement with the literature, menadione, H_2_O_2_, and t-BuOOH induced concentration-dependent decrements in the viability, motility, and progressive motility of spermatozoa (Figure 1). It is known from the literature that all of these induce intracellular OS processes, but regarding whether or not these intracellular changes can be measured with the Mioxsys system, we have had little information on this until now. Surprisingly, all of the applied treatments caused measurable changes in the ORP values. Namely, 1 mM and 2 mM H_2_O_2_ did not induce significant changes, but 5 mM caused a 1.42-fold decrement in the ORP level; 1 mM, 2 mM, and 5 mM menadione exposures resulted in 3.73-fold, 4.41-fold, and 5.02-fold increments in the ORP level, respectively; in the case of 1 mM t-BuOOH, no alteration could be observed, but 2 mM and 5 mM induced 1.25-fold and 1.57-fold increases (Figure 3). In all respects, menadione proved to be the most effective treatment. Changes in the sperm parameters and changes in the ORP values correlated with each other, which indicates that the method is suitable for the detection of intracellular ROS. In addition, the significant increments in the ORP after the menadione treatments suggest that there may have been much less SOD activity in the sperm, as it was published by O’Flaherty et al. [92] earlier. As a control, 5% DMSO was used, which did not appear to be toxic despite the high concentration, although it should be mentioned that in other experiments, 2% DMSO for 4 hours caused significant decreases in the viability and motility of human spermatozoa [97].

Ejaculate is a unique body fluid since it is only produced at the time of ejaculation. It consists of a cellular fraction (5%, spermatozoa, epithelial cells, leukocytes), secretions of the rete testis, epididymis, seminal vesicles, and the prostate (95%), and seminal plasma. The composition of the latter is highly complex and has several physiologic roles, which are crucial in fertilization, the final duty of spermatozoa. The largest portion (65–75%) of seminal plasma is secreted by the seminal vesicles containing a high amount of fructose. Fructose is the main energy source of the spermatozoa, providing their motility. Around 20–30% is produced by the prostate, it contains zinc (Zn) in large quantities, which has antibacterial properties, and has a role in the coagulation of semen along with zinc-binding proteins like *seminogelin I. Prostate specific antigen* (*PSA*) is another zinc-binding protein with a proteolytic effect responsible for the colliquation of semen, with widely known clinical importance in the screening and diagnosis of prostate cancer. The remaining proportions contain the secretions of the testis, epididymis, bulbourethral, and periurethral glands [44,98,99]. The neutral alfa glucosidase (NAG) is an epididymal enzyme with a still not clearly known function, but a role in spermatozoa maturation (modification of the membrane protein composition) and zona pellucida binding is probable [100]. The levels of NAG, fructose, and Zn can be measured and are important seminal markers. Their decreased levels can indicate inflammation or obstruction and give valuable information about the secretion status of the accessory glands. These biochemical assays are recommended by the actual, sixth version of The Laboratory Manual for the Examination and Processing of Human Semen provided by the WHO [101]. In the semen, the matured spermatozoa have a limited antioxidant capacity due to the loss of cytoplasm, which results in their particular vulnerability to OS. To protect the spermatozoa against various types of OS, the seminal plasma is present with a high antioxidant capacity provided by its non-protein antioxidant molecules and protein-based complex enzyme system. The ability of the seminal plasma to scavenge ROS can be characterized by its reductive potential; otherwise, the TAC which can be measured by various methods. Non-protein antioxidants (vitamin C (L-ascorbic acid), vitamin E (α-tocopherol), L-carnithine, zinc, selenium, co-enzyme Q10, etc.) are essential in the defensive mechanisms against OS, though their levels in the seminal plasma is diet-dependent, and therefore—assuming a healthy diet—more or less constant. Contrarily, the levels of the antioxidant enzymes CAT, SOD, GPx and glutathione reductase (GR), etc.) can be decreased or increased for the downregulation or upregulation of their expression [44,76,102]. As we can see, the seminal plasma has a role in a variety of processes crucial for successful fertilization: spermatozoa maturation, providing nutrients for motility, protection against bacteria or OS, and oocyte–spermatozoa interaction and fusion. In animals, it has been reported that even the negative effects of the cryopreservation–thawing process on motility, membrane integrity, and pregnancy rates can be attenuated with the supplementation of seminal plasma to the medium [103,104].

As can be seen in Figure 2 and Figure 4 (the elimination of seminal plasma resulted in more adverse effects of the applied treatments), seminal plasma had a great protective effect on sperm cells, thanks to its composition and antioxidant effects. For example, in the case of 1 mM, which was the least effective of all type of treatments, a much more negative effect was experienced. After the parallel H_2_O_2_ treatments, the viability and motility decreased by 2.15-fold and 16.68-fold, respectively. Similar decrements could also be measured in the cases of menadione and t-BuOOH. Once again, menadione proved to be the most effective treatment (Figure 2). Regarding the ORP measurements, 1 mM, 2 mM, and 5 mM H_2_O_2_, 1 mM, 2 mM, and 5 mM menadione, and 1 mM, 2 mM, and 5 mM t-BuOOH induced 1.00-fold, 1.09-fold, 1.12-fold, 1.23-fold, 1.27-fold, 1.27-fold, 1.20-fold, 1.40-fold, and 1.48-fold increments (Figure 4). In comparison to the raw semen samples, the initial ORP levels of the plasma-free samples were 6–7-fold higher, showed totally different patterns, indicating that the semen plasma had a more sophisticated composition than the PBS, and their composition and their conductivity differed. Seminal plasma serves as the main barrier against extracellular ROS, and the removal of the plasma abolishes this protection, as the intracellular activities of antioxidants are much lower [105]. In addition, although stronger effects were detected in the case of the plasma-free treatments (lower viability and motilities), this was not totally manifested in the ORP changes. The authors attribute this to the lower resolution of the device in the upper range.

One of the possible solutions for high ORP levels is antioxidant therapy. Antioxidants can play an important role in the treatment of male infertility. Used alone or in combination, the roles of vitamins A, C, and E, L-carnitine, N-acetylcysteine, coenzyme Q10, ω-3 fatty acid, and lycopene, as well as zinc, selenium, and folic acid should be highlighted. In 2018, a systematic review analyzing 26 studies found that antioxidant administration improved sperm parameters, sperm function, and ART outcomes, as well as live birth rates [106]. Similar results were also reported in the meta-analysis by Smith et al. [107], which included 61 studies with 6264 infertile men. In a meta-analysis of data from 23 studies on 1917 idiopathic male infertility patients treated with 10 different antioxidants, L-carnitine was found to be the most effective in improving sperm motility, and ω-3 fatty acid was found to be the most effective in improving sperm concentration, but there was no significant difference in pregnancy rates compared to placebo [108].

On the other hand, the Males, Antioxidants, and Infertility (MOXI) randomized trial found that 3 months of antioxidant treatment did not improve sperm parameters or DNA fragmentation compared to placebo in infertile men with male factor infertility [109]. Conversely, antioxidant treatment at a too-low dose or for too short a duration is ineffective, and overdosing can lead to equally harmful reductive stress [110,111]. Negative effects of antioxidant administration have also been reported, mainly in relation to the overuse of vitamin E [112]. Evidence on the role of antioxidant therapy in male infertility remains conflicting. A lot of the data come from low-quality RCTs, with a lack of placebo-controlled randomized trials, type of antioxidant used, and dose and duration of treatment [113]. Among other things, the above are the reasons why the WHO criteria do not contain specific protocols for the case of OS and the use of antioxidant treatments. Further studies are required.

## 4. Materials and Methods

### 4.1. Retrospective Analysis of ORP and Sperm Parameter Measurements

Our institute started to use the Mioxsys system (Caerus Biotech, Vilnius, Lithuania) in 2022. We performed a retrospective data analysis of previous measurements from this year to see how changes in the sperm count affected the other parameters (motility, progressive motility, viability, DNA fragmentation, normalized ORP level, and non-normalized ORP level). Semen analysis (concentration, motility, progressive motility, viability, and DNA fragmentation) was performed by an SCA SCOPE (Microptic S.L., Barcelona, Spain) automatic semen analysis system, and the ORP measurements were performed by the Mioxsys system [26]. In this period, the analyses of 183 semen samples were performed where the above-mentioned parameters were determined. There were not any exclusion criteria from the analysis.

### 4.2. Sample Collection for Treatments

After 3 days of abstinence, semen samples were collected by masturbation. After liquification in room temperature (not more than 1 h), the samples were divided into two groups. The first group had the raw semen samples and the second had the centrifugated and washed sperm cells. Namely, the samples were centrifugated at 400× *g* for 10 min. For washing, PBS was used, and the cell concentration was adjusted to 20 M/mL in PBS. In the case of the raw semen samples, the samples were centrifugated, and the cell numbers were adjusted with their own plasma. Every group contained 10–10 volunteers from our clinics. The inclusion criteria were the following: at least a 30 M/mL sperm count, 70% viability, maximum value of ORP of 1.36 mV/10^6^/mL to exclude existing OS, and at least 3.0 mL of semen to perform all of the treatments. If the volume allowed, parallel treatments were performed. Although WHO recommends 37 °C for liquefaction [101], which is the corporal temperature of the body of a woman where the semen will naturally reside after intercourse, based on some research, preparation in room temperature may result in better sperm parameters (vitality, motility, DNA fragmentation) [114].

### 4.3. Artificial ROS Induction

Menadione exposures were used to trigger O_2_^●−^ formation, H_2_O_2_ exposures were applied as a direct OS source, and t-BuOOH treatments were performed to cause the peroxidation of lipids [77,86,88]. The samples of both groups were aliquoted into Eppendorf tubes (300 μL/tube) and the following treatments were performed: (i) 0 mM, 1 mM, 2 mM, and 5 mM H_2_O_2_, (ii) 0 mM, 1 mM, 2 mM, and 5 mM menadione, (iii) 0 mM, 1 mM, 2 mM, and 5 mM t-BuOOH. For dilutions and control, DMSO was used, and its final volume was 5% in every case. All of the treatments were performed at 37 °C for two hours. Every 15 min, the samples were homogenized. After the treatments, samples were centrifugated at 400× *g* for 10 min and washed two times with PBS, except for the ORP determination of native samples.

### 4.4. Measurements of Basic Sperm Parameters and Their Oxido-Reduction State

Before the beginning of the exposures and after the two hours, the sperm motility, progressive motility, and viability were determined by an SCA SCOPE system based on the recommendations of the manufacturer.

For the quantification of induced OS, a Mioxsys system was used. Namely, a 30 μL sample was placed onto the oxido-reduction sensor, and the measured value was not divided by the cell concentration because the applied cell concentration was constant during the parallel treatments.

### 4.5. Statistical Analysis

The data were given as the average and deviation. A Shapiro–Wilks test was used to evaluate the distribution of the data. Non-normally distributed variables were examined using the non-parametric multiple tests of Mann–Whitney. GraphPad in Stat 7.0 software (Dotmatics, GraphPad Software Inc., Boston, MA, USA) was used for statistical analysis.

## 5. Conclusions

In conclusion, our results demonstrate the correlations among the ORP, count of sperm, viability, motility, and progressive motility. Based on our results, the acceptability of ORP can be problematic under 10 × 10^6^ sperm/mL and over 50 × 10^6^ sperm/mL. In order to clarify the exact effect of the sperm count on ORP acceptability, further investigations are needed.

With three different types of OS inducers, intracellular ROS formations were triggered. Namely, menadione exposures were used to induce O_2_^●−^, t-BuOOH lipid peroxidation was initiated, and for the third treatment, H_2_O_2_ was applied. All of the applied treatments induced OS in spermatozoa in a concentration-dependent manner, and it was manifested in reduced viability, motility, and progressive motility. Considerable changes were experienced in the ORP levels after the treatments, indicating OS in sperm cells. Approximately 95% of ejaculate is seminal plasma containing substances with antioxidant properties. After the elimination of the seminal plasma, the applied treatments exerted more serious negative effects on the parameters of spermatozoa.

In the future, the ORP measurement of semen could be a useful method for OS determination in andrology. It is fast, user-friendly, and does not require expensive devices or special expertise. The only disadvantages are the expensive consumable (as a result of this, the main limitation of our work is the limited number of treatments), and the uncertain acceptability of the results in cases of viscous samples and samples with very low or very high sperm counts. It should be considered that under or above a certain cell number, the method is unnecessary. To clarify these problems, further experiments are suggested.

## Figures and Tables

**Table 1:** Distribution of semen parameters based on the concentration. ** *p* < 1%; *** *p* < 0.1%. *p* values were calculated via the Mann–Whitney test.

	n	Concentration M/mL	Motility %	Progressive Motility %	Viability %	ORP mV/10^6^/mL	ORP mV	DNA Fragmentation %
<16 M/mL	69	8.3 ± 4.1	25.8 ± 14.1	19.7 ± 13.1	60.5 ± 8.7	8.5 ± 13.9	38.5 ± 16.6	20.5 ± 10.6
>16 M/mL	90	61.6 ± 58.8 ***	37.4 ± 17.8 ***	28.8 ± 15.8 ***	67.4 ± 11.6 ***	1.0 ± 0.9 **	40.6 ± 25.5	16.8 ± 10.0

## Data Availability

Not applicable.

## References

[1] Thonneau P., Marchand S., Tallec A., Ferial M.L., Ducot B., Lansac J., Lopes P., Tabaste J.M., Spira A. (1991). Incidence and Main Causes of Infertility in a Resident Population (1,850,000) of Three French Regions (1988–1989). Hum. Reprod..

[2] Hirsh A. (2003). Male Subfertility. BMJ.

[3] Siddiq F.M., Sigman M. (2002). A New Look at the Medical Management of Infertility. Urol. Clin. N. Am..

[4] Greenberg S.H., Lipshultz L.I., Wein A.J. (1978). Experience with 425 Subfertile Male Patients. J. Urol..

[5] Hamada A., Esteves S.C., Nizza M., Agarwal A. (2012). Unexplained Male Infertility: Diagnosis and Management. Int. Braz. J. Urol..

[6] Agarwal A., Parekh N., Panner Selvam M.K., Henkel R., Shah R., Homa S.T., Ramasamy R., Ko E., Tremellen K., Esteves S. (2019). Male Oxidative Stress Infertility (MOSI): Proposed Terminology and Clinical Practice Guidelines for Management of Idiopathic Male Infertility. World J. Men’s Health.

[7] Saalu L.C. (2010). The Incriminating Role of Reactive Oxygen Species in Idiopathic Male Infertility: An Evidence Based Evaluation. Pakistan J. Biol. Sci. PJBS.

[8] Iommiello V.M., Albani E., Di Rosa A., Marras A., Menduni F., Morreale G., Levi S.L., Pisano B., Levi-Setti P.E. (2015). Ejaculate Oxidative Stress Is Related with Sperm DNA Fragmentation and Round Cells. Int. J. Endocrinol..

[9] Ozmen B., Koutlaki N., Youssry M., Diedrich K., Al-Hasani S. (2007). DNA Damage of Human Spermatozoa in Assisted Reproduction: Origins, Diagnosis, Impacts and Safety. Reprod. Biomed. Online.

[10] O’Flaherty C., Scarlata E. (2022). OXIDATIVE STRESS AND REPRODUCTIVE FUNCTION: The Protection of Mammalian Spermatozoa against Oxidative Stress. Reproduction.

[11] Jones R., Mann T., Sherins R. (1979). Peroxidative Breakdown of Phospholipids in Human Spermatozoa, Spermicidal Properties of Fatty Acid Peroxides, and Protective Action of Seminal Plasma. Fertil. Steril..

[12] Kowalczyk A. (2022). The Role of the Natural Antioxidant Mechanism in Sperm Cells. Reprod. Sci..

[13] Smith T.B., Dun M.D., Smith N.D., Curry B.J., Connaughton H.S., Aitken R.J. (2013). The Presence of a Truncated Base Excision Repair Pathway in Human Spermatozoa That Is Mediated by OGG1. J. Cell Sci..

[14] Sabeti P., Pourmasumi S., Rahiminia T., Akyash F., Talebi A.R. (2016). Etiologies of Sperm Oxidative Stress. Int. J. Reprod. Biomed..

[15] Aitken R.J., Baker H.W.G. (1995). Andrology: Seminal Leukocytes: Passengers, Terrorists or Good Samaritans?. Hum. Reprod..

[16] Aitken R.J. (2017). Reactive Oxygen Species as Mediators of Sperm Capacitation and Pathological Damage. Mol. Reprod. Dev..

[17] Fisher H.M., Aitken R.J. (1997). Comparative Analysis of the Ability of Precursor Germ Cells and Epididymal Spermatozoa to Generate Reactive Oxygen Metabolites. J. Exp. Zool..

[18] O’Flaherty C., de Lamirande E., Gagnon C. (2006). Positive Role of Reactive Oxygen Species in Mammalian Sperm Capacitation: Triggering and Modulation of Phosphorylation Events. Free Radic. Biol. Med..

[19] Ávila C., Vinay J.I., Arese M., Saso L., Rodrigo R. (2022). Antioxidant Intervention against Male Infertility: Time to Design Novel Strategies. Biomedicines.

[20] Aitken R.J. (1999). The Amoroso Lecture. The Human Spermatozoon—A Cell in Crisis?. J. Reprod. Fertil..

[21] Du Plessis S.S., Agarwal A., Halabi J., Tvrda E. (2015). Contemporary Evidence on the Physiological Role of Reactive Oxygen Species in Human Sperm Function. J. Assist. Reprod. Genet..

[22] Agarwal A., Majzoub A. (2017). Laboratory Tests for Oxidative Stress. Indian J. Urol..

[23] Agarwal A., Virk G., Ong C., du Plessis S.S. (2014). Effect of Oxidative Stress on Male Reproduction. World J. Men’s Health.

[24] Lampiao F. (2012). Free Radicals Generation in an in Vitro Fertilization Setting and How to Minimize Them. World J. Obstet. Gynecol..

[25] Agarwal A., Roychoudhury S., Bjugstad K.B., Cho C.-L. (2016). Oxidation-Reduction Potential of Semen: What Is Its Role in the Treatment of Male Infertility?. Ther. Adv. Urol..

[26] Castleton P., Gyawali P., Mathews N., Mutuku S.M., Sharkey D.J., McPherson N.O. (2022). MiOXSYS(^®^) and OxiSperm(^®^) II Assays Appear to Provide No Clinical Utility for Determining Oxidative Stress in Human Sperm—Results from Repeated Semen Collections. Andrology.

[27] Martemucci G., Costagliola C., Mariano M., D’andrea L., Napolitano P., D’Alessandro A.G. (2022). Free Radical Properties, Source and Targets, Antioxidant Consumption and Health. Oxygen.

[28] Butterfield D.A., Griffin S., Munch G., Pasinetti G.M. (2002). Amyloid Beta-Peptide and Amyloid Pathology Are Central to the Oxidative Stress and Inflammatory Cascades under Which Alzheimer’s Disease Brain Exists. J. Alzheimer’s Dis..

[29] Schapira A.H.V. (2008). Mitochondria in the Aetiology and Pathogenesis of Parkinson’s Disease. Lancet Neurol..

[30] Mhatre M., Floyd R.A., Hensley K. (2004). Oxidative Stress and Neuroinflammation in Alzheimer’s Disease and Amyotrophic Lateral Sclerosis: Common Links and Potential Therapeutic Targets. J. Alzheimer’s Dis..

[31] Baradaran A., Nasri H., Rafieian-Kopaei M. (2014). Oxidative Stress and Hypertension: Possibility of Hypertension Therapy with Antioxidants. J. Res. Med. Sci. Off. J. Isfahan Univ. Med. Sci..

[32] Teuber J.P., Essandoh K., Hummel S.L., Madamanchi N.R., Brody M.J. (2022). NADPH Oxidases in Diastolic Dysfunction and Heart Failure with Preserved Ejection Fraction. Antioxidants.

[33] Nomani H., Bayat G., Sahebkar A., Fazelifar A.F., Vakilian F., Jomezade V., Johnston T.P., Mohammadpour A.H. (2019). Atrial Fibrillation in β-Thalassemia Patients with a Focus on the Role of Iron-Overload and Oxidative Stress: A Review. J. Cell. Physiol..

[34] Bashan N., Kovsan J., Kachko I., Ovadia H., Rudich A. (2009). Positive and Negative Regulation of Insulin Signaling by Reactive Oxygen and Nitrogen Species. Physiol. Rev..

[35] Fernández-Sánchez A., Madrigal-Santillán E., Bautista M., Esquivel-Soto J., Morales-González A., Esquivel-Chirino C., Durante-Montiel I., Sánchez-Rivera G., Valadez-Vega C., Morales-González J.A. (2011). Inflammation, Oxidative Stress, and Obesity. Int. J. Mol. Sci..

[36] Daenen K., Andries A., Mekahli D., Van Schepdael A., Jouret F., Bammens B. (2019). Oxidative Stress in Chronic Kidney Disease. Pediatr. Nephrol..

[37] Liu W., Gao Y., Li H., Wang X., Jin M., Shen Z., Yang D., Zhang X., Wei Z., Chen Z. (2023). Association between Oxidative Stress, Mitochondrial Function of Peripheral Blood Mononuclear Cells and Gastrointestinal Cancers. J. Transl. Med..

[38] Valavanidis A., Vlachogianni T., Fiotakis K., Loridas S. (2013). Pulmonary Oxidative Stress, Inflammation and Cancer: Respirable Particulate Matter, Fibrous Dusts and Ozone as Major Causes of Lung Carcinogenesis through Reactive Oxygen Species Mechanisms. Int. J. Environ. Res. Public Health.

[39] Kalinina E.V., Gavriliuk L.A., Pokrovsky V.S. (2022). Oxidative Stress and Redox-Dependent Signaling in Prostate Cancer. Biochemistry.

[40] Gurer-Orhan H., Ince E., Konyar D., Saso L., Suzen S. (2018). The Role of Oxidative Stress Modulators in Breast Cancer. Curr. Med. Chem..

[41] Spector A. (1995). Oxidative Stress-Induced Cataract: Mechanism of Action. FASEB J. Off. Publ. Fed. Am. Soc. Exp. Biol..

[42] Aitken R.J., West K., Buckingham D. (1994). Leukocytic Infiltration into the Human Ejaculate and Its Association with Semen Quality, Oxidative Stress, and Sperm Function. J. Androl..

[43] Aitken R.J. (1994). A Free Radical Theory of Male Infertility. Reprod. Fertil. Dev..

[44] Ribeiro J.C., Braga P.C., Martins A.D., Silva B.M., Alves M.G., Oliveira P.F. (2021). Antioxidants Present in Reproductive Tract Fluids and Their Relevance for Fertility. Antioxidants.

[45] Salonia C.A., Bettocchi P., Capogrosso J., Carvalho G., Corona G., Hatzichristodoulou T.H., Jones A., Kadioglu J.I., Martinez-Salamanca S., Minhas E.C. (2023). EAU Guidelines on Sexual and Reproductive Health.

[46] Colpi G.M., Francavilla S., Haidl G., Link K., Behre H.M., Goulis D.G., Krausz C., Giwercman A. (2018). European Academy of Andrology Guideline Management of Oligo-Astheno-Teratozoospermia. Andrology.

[47] Bender Atik R., Christiansen O.B., Elson J., Kolte A.M., Lewis S., Middeldorp S., Nelen W., Peramo B., Quenby S., Vermeulen N. (2018). ESHRE Guideline: Recurrent Pregnancy Loss. Hum. Reprod. Open.

[48] Harlev A., Agarwal A., Gunes S.O., Shetty A., du Plessis S.S. (2015). Smoking and Male Infertility: An Evidence-Based Review. World J. Men’s Health.

[49] Maneesh M., Jayalekshmi H., Dutta S., Chakrabarti A., Vasudevan D.M. (2005). Effect of Chronic Ethanol Administration on Testicular Antioxidant System and Steroidogenic Enzyme Activity in Rats. Indian J. Exp. Biol..

[50] Abarikwu S.O., Duru Q.C., Chinonso O.V., Njoku R.-C. (2016). Antioxidant Enzymes Activity, Lipid Peroxidation, Oxidative Damage in the Testis and Epididymis, and Steroidogenesis in Rats after Co-Exposure to Atrazine and Ethanol. Andrologia.

[51] Gautam R., Singh K.V., Nirala J., Murmu N.N., Meena R., Rajamani P. (2019). Oxidative Stress-Mediated Alterations on Sperm Parameters in Male Wistar Rats Exposed to 3G Mobile Phone Radiation. Andrologia.

[52] Kesari K.K., Behari J. (2012). Evidence for Mobile Phone Radiation Exposure Effects on Reproductive Pattern of Male Rats: Role of ROS. Electromagn. Biol. Med..

[53] Erogul O., Oztas E., Yildirim I., Kir T., Aydur E., Komesli G., Irkilata H.C., Irmak M.K., Peker A.F. (2006). Effects of Electromagnetic Radiation from a Cellular Phone on Human Sperm Motility: An in Vitro Study. Arch. Med. Res..

[54] Mortazavi S.M.J., Tavassoli A., Ranjbari F., Moammaiee P. (2010). Effects of Laptop Computers’ Electromagnetic Field on Sperm Quality. J. Reprod. Fertil..

[55] Zhang G., Jiang F., Chen Q., Yang H., Zhou N., Sun L., Zou P., Yang W., Cao J., Zhou Z. (2020). Associations of Ambient Air Pollutant Exposure with Seminal Plasma MDA, Sperm MtDNA Copy Number, and MtDNA Integrity. Environ. Int..

[56] Li D., Yin D., Han X. (2007). Methyl Tert-Butyl Ether (MTBE)-Induced Cytotoxicity and Oxidative Stress in Isolated Rat Spermatogenic Cells. J. Appl. Toxicol..

[57] Leisegang K., Sengupta P., Agarwal A., Henkel R. (2021). Obesity and Male Infertility: Mechanisms and Management. Andrologia.

[58] Wang K., Gao Y., Wang C., Liang M., Liao Y., Hu K. (2022). Role of Oxidative Stress in Varicocele. Front. Genet..

[59] Lee J.-D., Jeng S.-Y., Lee T.-H. (2006). Increased Expression of Hypoxia-Inducible Factor-1alpha in the Internal Spermatic Vein of Patients with Varicocele. J. Urol..

[60] Agarwal A., Hamada A., Esteves S.C. (2012). Insight into Oxidative Stress in Varicocele-Associated Male Infertility: Part 1. Nat. Rev. Urol..

[61] Nago M., Arichi A., Omura N., Iwashita Y., Kawamura T., Yumura Y. (2021). Aging Increases Oxidative Stress in Semen. Investig. Clin. Urol..

[62] Aitken R.J., Harkiss D., Knox W., Paterson M., Irvine D.S. (1998). A Novel Signal Transduction Cascade in Capacitating Human Spermatozoa Characterised by a Redox-Regulated, CAMP-Mediated Induction of Tyrosine Phosphorylation. J. Cell Sci..

[63] Davis B.K. (1981). Timing of Fertilization in Mammals: Sperm Cholesterol/Phospholipid Ratio as a Determinant of the Capacitation Interval. Proc. Natl. Acad. Sci. USA.

[64] Visconti P.E., Bailey J.L., Moore G.D., Pan D., Olds-Clarke P., Kopf G.S. (1995). Capacitation of Mouse Spermatozoa. I. Correlation between the Capacitation State and Protein Tyrosine Phosphorylation. Development.

[65] Agarwal A., Qiu E., Sharma R. (2018). Laboratory Assessment of Oxidative Stress in Semen. Arab J. Urol..

[66] Kashou A.H., Sharma R., Agarwal A. (2013). Assessment of Oxidative Stress in Sperm and Semen. Methods Mol. Biol..

[67] Aitken R.J., Buckingham D.W., West K.M. (1992). Reactive Oxygen Species and Human Spermatozoa: Analysis of the Cellular Mechanisms Involved in Luminol- and Lucigenin-Dependent Chemiluminescence. J. Cell. Physiol..

[68] Aitken R.J., Buckingham D. (1992). Enhanced Detection of Reactive Oxygen Species Produced by Human Spermatozoa with 7-Dimethyl Amino-Naphthalin-1, 2-Dicarbonic Acid Hydrazide. Int. J. Androl..

[69] Agarwal A., Ahmad G., Sharma R. (2015). Reference Values of Reactive Oxygen Species in Seminal Ejaculates Using Chemiluminescence Assay. J. Assist. Reprod. Genet..

[70] Kobayashi H., Gil-Guzman E., Mahran A.M., Nelson D.R., Thomas A.J.J., Agarwa A., Rakesh (2001). Quality Control of Reactive Oxygen Species Measurement by Luminol-Dependent Chemiluminescence Assay. J. Androl..

[71] Garcia-Segura S., Ribas-Maynou J., Lara-Cerrillo S., Garcia-Peiró A., Castel A.B., Benet J., Oliver-Bonet M. (2020). Relationship of Seminal Oxidation-Reduction Potential with Sperm DNA Integrity and PH in Idiopathic Infertile Patients. Biology.

[72] Henkel R., Morris A., Vogiatzi P., Saleh R., Sallam H., Boitrelle F., Garrido N., Arafa M., Gül M., Rambhatla A. (2022). Predictive Value of Seminal Oxidation-Reduction Potential Analysis for Reproductive Outcomes of ICSI. Reprod. Biomed. Online.

[73] Gille G., Sigler K. (1995). Oxidative Stress and Living Cells. Folia Microbiol..

[74] Sarniak A., Lipińska J., Tytman K., Lipińska S. (2016). Endogenous Mechanisms of Reactive Oxygen Species (ROS) Generation. Postepy Hig. Med. Dosw..

[75] Valko M., Rhodes C.J., Moncol J., Izakovic M., Mazur M. (2006). Free Radicals, Metals and Antioxidants in Oxidative Stress-Induced Cancer. Chem. Biol. Interact..

[76] Ayad B., Omolaoye T.S., Louw N., Ramsunder Y., Skosana B.T., Oyeipo P.I., Du Plessis S.S. (2022). Oxidative Stress and Male Infertility: Evidence from a Research Perspective. Front. Reprod. Health.

[77] Fukui M., Choi H.J., Zhu B.T. (2012). Rapid Generation of Mitochondrial Superoxide Induces Mitochondrion-Dependent but Caspase-Independent Cell Death in Hippocampal Neuronal Cells That Morphologically Resembles Necroptosis. Toxicol. Appl. Pharmacol..

[78] Halliwell B., Gutteridge J.M.C. (1999). Free Radicals in Biology and Medicine.

[79] Aitken R.J., Smith T.B., Lord T., Kuczera L., Koppers A.J., Naumovski N., Connaughton H., Baker M.A., De Iuliis G.N. (2013). On Methods for the Detection of Reactive Oxygen Species Generation by Human Spermatozoa: Analysis of the Cellular Responses to Catechol Oestrogen, Lipid Aldehyde, Menadione and Arachidonic Acid. Andrology.

[80] Zhu Z., Zeng Y., Zeng W. (2022). Cysteine Improves Boar Sperm Quality via Glutathione Biosynthesis during the Liquid Storage. Anim. Biosci..

[81] Guthrie H.D., Welch G.R., Long J.A. (2008). Mitochondrial Function and Reactive Oxygen Species Action in Relation to Boar Motility. Theriogenology.

[82] Goodla L., Morrell J.M., Yusnizar Y., Stålhammar H., Johannisson A. (2014). Quality of Bull Spermatozoa after Preparation by Single-Layer Centrifugation. J. Dairy Sci..

[83] Saran M., Bors W. (1991). Direct and Indirect Measurements of Oxygen Radicals. Klin. Wochenschr..

[84] Oya Y., Yamamoto K. (1988). The Biological Activity of Hydrogen Peroxide. IV. Enhancement of Its Clastogenic Actions by Coadministration of L-Histidine. Mutat. Res..

[85] Chaki S.P., Misro M.M. (2002). Assessment of Human Sperm Function after Hydrogen Peroxide Exposure. Development of a Vaginal Contraceptive. Contraception.

[86] Pujianto D.A., Oktarina M., Sharma Sharaswati I.A., Yulhasri (2021). Hydrogen Peroxide Has Adverse Effects on Human Sperm Quality Parameters, Induces Apoptosis, and Reduces Survival. J. Hum. Reprod. Sci..

[87] Siddiqui R.A., Harvey K., Stillwell W. (2008). Anticancer Properties of Oxidation Products of Docosahexaenoic Acid. Chem. Phys. Lipids.

[88] Girotti A.W. (1998). Lipid Hydroperoxide Generation, Turnover, and Effector Action in Biological Systems. J. Lipid Res..

[89] Halliwell B., Chirico S. (1993). Lipid Peroxidation: Its Mechanism, Measurement, and Significance. Am. J. Clin. Nutr..

[90] Wang G., Hong Y., Johnson M.K., Maier R.J. (2006). Lipid Peroxidation as a Source of Oxidative Damage in Helicobacter Pylori: Protective Roles of Peroxiredoxins. Biochim. Biophys. Acta.

[91] Howlett N.G., Avery S. (1997). V Induction of Lipid Peroxidation during Heavy Metal Stress in Saccharomyces Cerevisiae and Influence of Plasma Membrane Fatty Acid Unsaturation. Appl. Environ. Microbiol..

[92] O’Flaherty C. (2018). Peroxiredoxin 6: The Protector of Male Fertility. Antioxidants.

[93] Moazamian R., Polhemus A., Connaughton H., Fraser B., Whiting S., Gharagozloo P., Aitken R.J. (2015). Oxidative Stress and Human Spermatozoa: Diagnostic and Functional Significance of Aldehydes Generated as a Result of Lipid Peroxidation. Mol. Hum. Reprod..

[94] Aboua Y., Brooks N. (2009). Impact of Organic Hydroperoxides on Rat Testicular Tissue and Epididymal Sperm. Afr. J. Biotechnol..

[95] Kumar T.R., Muralidhara (2007). Induction of Oxidative Stress by Organic Hydroperoxides in Testis and Epididymal Sperm of Rats in Vivo. J. Androl..

[96] Wu P.Y., Scarlata E., O’Flaherty C. (2020). Long-Term Adverse Effects of Oxidative Stress on Rat Epididymis and Spermatozoa. Antioxidants.

[97] Bisconti M., Grosjean P., Arcolia V., Simon J.-F., Hennebert E. (2023). Influence of Two Widely Used Solvents, Ethanol and Dimethyl Sulfoxide, on Human Sperm Parameters. Int. J. Mol. Sci..

[98] Nieschlag E., Behre H.M., Nieschlag S. (2010). Andrology: Male Reproductive Health and Dysfunction.

[99] Juyena N.S., Stelletta C. (2012). Seminal Plasma: An Essential Attribute to Spermatozoa. J. Androl..

[100] Ben Ali H., Guerin J.F., Pinatel M.C., Mathieu C., Boulieu D., Tritar B. (1994). Relationship between Semen Characteristics, Alpha-Glucosidase and the Capacity of Spermatozoa to Bind to the Human Zona Pellucida. Int. J. Androl..

[101] World Health Organization (2021). WHO Laboratory Manual for the Examination and Processing of Human Semen.

[102] Gupta S., Finelli R., Agarwal A., Henkel R. (2021). Total Antioxidant Capacity-Relevance, Methods and Clinical Implications. Andrologia.

[103] Heise A., Kähn W., Volkmann D.H., Thompson P.N., Gerber D. (2010). Influence of Seminal Plasma on Fertility of Fresh and Frozen-Thawed Stallion Epididymal Spermatozoa. Anim. Reprod. Sci..

[104] Hernández M., Roca J., Calvete J.J., Sanz L., Muiño-Blanco T., Cebrián-Pérez J.A., Vázquez J.M., Martínez E.A. (2007). Cryosurvival and in Vitro Fertilizing Capacity Postthaw Is Improved When Boar Spermatozoa Are Frozen in the Presence of Seminal Plasma from Good Freezer Boars. J. Androl..

[105] Castleton P.E., Deluao J.C., Sharkey D.J., McPherson N.O. (2022). Measuring Reactive Oxygen Species in Semen for Male Preconception Care: A Scientist Perspective. Antioxidants.

[106] Majzoub A., Agarwal A. (2018). Systematic Review of Antioxidant Types and Doses in Male Infertility: Benefits on Semen Parameters, Advanced Sperm Function, Assisted Reproduction and Live-Birth Rate. Arab J. Urol..

[107] Smits R.M., Mackenzie-Proctor R., Yazdani A., Stankiewicz M.T., Jordan V., Showell M.G. (2019). Antioxidants for Male Subfertility. Cochrane Database Syst. Rev..

[108] Li K.-P., Yang X.-S., Wu T. (2022). The Effect of Antioxidants on Sperm Quality Parameters and Pregnancy Rates for Idiopathic Male Infertility: A Network Meta-Analysis of Randomized Controlled Trials. Front. Endocrinol..

[109] Steiner A.Z., Hansen K.R., Barnhart K.T., Cedars M.I., Legro R.S., Diamond M.P., Krawetz S.A., Usadi R., Baker V.L., Coward R.M. (2020). The Effect of Antioxidants on Male Factor Infertility: The Males, Antioxidants, and Infertility (MOXI) Randomized Clinical Trial. Fertil. Steril..

[110] Henkel R., Sandhu I.S., Agarwal A. (2019). The Excessive Use of Antioxidant Therapy: A Possible Cause of Male Infertility?. Andrologia.

[111] Kopa Z., Keszthelyi M., Sofikitis N. (2021). Administration of Antioxidants in the Infertile Male: When It May Have a Beneficial Effect?. Curr. Pharm. Des..

[112] Ménézo Y.J.R., Hazout A., Panteix G., Robert F., Rollet J., Cohen-Bacrie P., Chapuis F., Clément P., Benkhalifa M. (2007). Antioxidants to Reduce Sperm DNA Fragmentation: An Unexpected Adverse Effect. Reprod. Biomed. Online.

[113] Agarwal A., Leisegang K., Majzoub A., Henkel R., Finelli R., Panner Selvam M.K., Tadros N., Parekh N., Ko E.Y., Cho C.L. (2021). Utility of Antioxidants in the Treatment of Male Infertility: Clinical Guidelines Based on a Systematic Review and Analysis of Evidence. World J. Men’s Health.

[114] Thijssen A., Klerkx E., Huyser C., Bosmans E., Campo R., Ombelet W. (2014). Influence of Temperature and Sperm Preparation on the Quality of Spermatozoa. Reprod. Biomed. Online.

